# Impact of COVID-19 on Malaysian dental students’ physical, mental, financial and academic concerns

**DOI:** 10.1186/s12903-022-02081-w

**Published:** 2022-02-23

**Authors:** Widya Lestari, Nur Hazirah Yazid, Zawin Najah Azhar, Azlini Ismail, Cortino Sukotjo

**Affiliations:** 1 Fundamental Dental and Medical Sciences Department, Kulliyyah of Dentistry, International Islamic University Malaysia, Kuantan, Malaysia; 2 Kulliyyah of Dentistry, International Islamic University Malaysia, Kuantan, Malaysia; 3 Department of Restorative Dentistry, University of Illinois at Chicago, Chicago, USA

**Keywords:** Dental education, COVID-19, Mental health, Pandemic, Dentistry, Medicine

## Abstract

**Background:**

The coronavirus disease 2019 (COVID-19) caused by a novel coronavirus (SARS-CoV-2) has gained worldwide attention and proved to hold an impact to humankind in all aspects of life. Dental students’ performances may indirectly be affected following the preventive measures in containing the disease. This study aims to evaluate the impact of COVID-19 pandemic on physical, mental, financial health and academic concern among dental students in Malaysia.

**Methods:**

The current research implemented a cross sectional study among dental students in Malaysia. Assessment of the impact of COVID-19 on dental education was done by the distribution of a set of online survey consisting of 28 questions to dental students (n = 353) from public and private universities in Malaysia. The questionnaires include sociodemographic backgrounds and assessment on the mental health, financial health, physical health and academic concern. Kruskal Wallis test and Mann–Whitney U test were used to analyse the impact of COVID-19 to these 4 domains according to sociodemographic background.

**Results:**

A total number of 353 respondents was recorded and 76.2% comprised of female. 59.7% were clinical students and 40.3% were preclinical students. Most of students were concerned about their own emotional health, financial concern, physical wellbeing, in which Year 3 students were found to be more concerned about their mental and financial health concern.

**Conclusions:**

COVID-19 pandemic had indeed significantly affected Malaysian dental students mainly due to fear of the quality of online learning and the amount of clinical skills acquired. Therefore, it is important to identify dental stressors and lessen the impact of COVID-19 to dental students.

## Background

In 2019, a disease outbreak was reported in Wuhan, China [1]. The outbreak was attributed to a novel coronavirus and named the Coronavirus disease 2019 (COVID-19). Within a few months, the outbreak had spread across the globe at unprecedented speed [2], prompting the World Health Organization (WHO) to declare it a public health emergency of international concern on the 30th January 2020 [3].

In Malaysia, the first case of COVID-19 was detected in January 2020 and was traced back to 3 Chinese nationals who travelled into Malaysia via Singapore and had come into close contact with an infected person in Singapore [4]. The Ministry of Health Malaysia immediately devised standard national guidelines for the management of COVID-19. Preventive measures covering Movement Control Orders (MCO) and Standard Operating Procedures (SOP) in public were implemented with almost immediate effect to contain the spread of COVID-19, including prohibition of mass gatherings, closure of government and private premises, and closure of public and private education institutions at all levels nationwide [4].

According to Hung et al. [5], global efforts to mitigate the spread of COVID-19 left no aspect of daily life unaffected. University students were not left out, as many educational institutions were forced to close their campuses and transitioned from in-person to online learning. Dental students in particular were massively affected as the United States Occupational Safety and Health Administration placed persons involved in dental practice and education in the very high-risk category for viral exposure and disease transmission due to close contact to patients during practice and generation of aerosol droplets during aerosol-generating dental procedures (AGP) in the clinic. The main challenges identified in dental education include preserving the health and safety of students, faculty and staff, ensuring the continuity and quality of dental education, securing confidence in health and safety measures and keeping up with changing health updates and guidelines. Other challenges include the suspension of clinical teaching in educational institutions, implementation of stay-at-home policies and enforcement of social distancing practices in laboratories and the clinic, which may affect the competency of the graduating student [6].

Dental students and practitioners in particular have been placed at an increased risk category for contracting the virus due to the nature of dental practice that exposes students and practitioners to bodily fluids from the patient, including blood and saliva, and the predominant mode of transmission of the virus being via contact with respiratory droplets from an infected individual [5]. The implementation of strict measures such as lockdowns, isolation, distancing, wearing of masks, transition into online remote teaching and delays in resuming normal education activities have helped to contain the spread of the COVID-19 outbreak in Malaysia. However, these measures have caused undeniable, significant impacts on the socio-psychological and mental well-being of the population, including dental students [2].

To date, several studies have been conducted on the mental health impact of the COVID-19 pandemic on persons involved in dental practice- from students to dental academic staff, to practitioners. However, most of this research has been limited to subjects in China and in the West. Few studies have investigated the mental health impact of the COVID-19 pandemic in Malaysia [2, 7]. To date, no studies have assessed the physical, mental, financial, and academic impacts of the pandemic on dental students in Malaysia. According to Malaysian Dental Council, from the 13 dental institutions in Malaysia, a grand total of 3513 dental students were accounted for from both public and private universities, hence Malaysian dental students were not exempt from the impacts of COVID-19. All 13 dental universities were in Peninsular Malaysia with 5 of them located at Kuala Lumpur. Dental institutions in Malaysia practice a 5-year undergraduate dental education programme. The first 2 years dental students (Year 1 and Year 2) are focused on didactic education and laboratory course (preclinical), while the last 3 years dental students (Year 3, Year 4 and Year 5) aimed on clinical training [8]. Closure of institutions, including clinics and laboratory, shifting of in-person to virtual learning and limited clinical practice and exposure following Movement Control Order (MCO) implemented by Malaysia government had greatly affected the dental students’ performances.

Thus, the main objective of this study is to evaluate the impact of the COVID-19 pandemic on dental education with a focus on physical, mental, financial and academic concerns respectively, in Malaysian dental students. The findings from this study should provide vital information on the impact of COVID-19 to dental students in Malaysia to allow proper intervention measures to be carried out under the current pandemic, as well as improve preparedness for future pandemic outbreaks.

## Methods

### Study population

An exploratory study using a cross-sectional online survey in English medium was carried out to investigate how COVID-19 impacts dental education in Malaysia. The survey was carried out from March 12 till April 21, 2021. The recommended sample size for this study is 347 which was calculated by using Raosoft software (http://www.raosoft.com/samplesize.html) with 95% confidence interval and 5% acceptable margin of error. Participants were invited from all dental schools in Malaysia. A Google Form survey invitation was circulated to all students’ representatives from each dental schools in Malaysia via WhatsApp messaging and email with reminders sent periodically to the deans from respective dental schools. Participants signed up for the study voluntarily and consent was obtained prior to the start of the survey. The response of the participants was kept confidential.

### Survey instrument

Hung’s questionnaire was used to determine the impact of COVID-19 on dental education in Malaysia [5]. Some items were modified to suit Malaysian student conditions after face to face validation and removed after a reliability test done during pilot study. Survey items focused on socio-demographic background of the participants, with 4 general domains: (1) Academic performance, (2) Physical health, (3) Mental health, and (4) Financial health. A total of 28 items related to educational experience and physical, mental, and financial health were included in the final survey.

Socio-demographic background consists of 8 items including gender, age, dental school, year of study, race, citizenship, current mode of living and living condition.

The academic performance domain consisted of 11 items with 4 items describing respondents’ concerns about clinical performance and their likelihood to pass the examinations in a timely manner. Response was rated using a five-point Likert Scale ranging between 1 (Extremely not concerned) to 5 (Extremely concerned). Some questions that covered emotional aspects, such as finding it difficult to be motivated in study was also rated by the Likert Scale ranging between 1 (Never) and 5 (Always).

The physical health concern domain was assessed based on 5 items including respondents’ general concerns for their physical health, as well as their concerns about getting infected by COVID-19 when interacting with people during class attendance. Responses were rated using a five-point Likert Scale ranging between 1(Extremely not concerned) and 5 (Extremely concerned).

The mental health concern domain consisted of 8 items which included emotional aspects such as feeling anxious about the future, feeling stressed and feeling depressed. The responses were rated on the Likert Scale ranging between 1 (Never) and 5 (Always).

The financial health concern domain consisted of 4 items that assessed the financial preparation and stability of the respondents for the COVID-19 pandemic. Three items were rated using the five-point Likert Scale ranging between 1 (Never) and 5 (Always) and 1 item was rated using the five-point Likert Scale ranging between 1 (Extremely not concerned) and 5 (Extremely concerned) respectively.

### Validity and reliability of questionnaires

Face-to-face validation process was conducted on five students from Kulliyyah of Dentistry, International Islamic University Malaysia (IIUM). The respondents were required to answer the questionnaires and were asked to evaluate the questionnaires in terms of readability and wording. A pilot study was carried out among 40 dental students to test for the reliability of questionnaires using Cronbach’s Alpha. Final set of questionnaires we used in the study consists of 28 items out of 32 items (4 items were removed from the original study) had Cronbach’s Alpha value more than 0.8 for all domains tested.

### Data analysis

Data analysis was carried out using IBM SPSS version 25 software (IBM Corporation, Armonk, NY, USA). Descriptive statistics was used to determine the distribution of the item responses, with continuous variables reported as mean and standard deviation (SD). The impact of COVID-19 on mental health concern, physical health concern, financial concern, academic concern was analysed according to the sociodemographic backgrounds (gender, year of study, current mode and living condition by using non parametric tests which are Mann – Whitney U test for comparing two groups and Kruskal Wallis test for comparing three and more groups, as the data was not normally distributed.

## Results

### Demographic characteristics

A total of 353 respondents from all dental schools in Malaysia submitted the completed online questionnaire assessing the impact of COVID-19 on dental students. Majority of the respondents were from IIUM (45.6%) followed by AIMST University (13.9%) and UM (11.3%). The questionnaire consists of 28 questions comprising 4 sections covering mental health concerns, physical health concerns, financial concerns and academic concerns. The demographic information of the participants is presented in Table 1. Out of 353 participants, 76.2% were female while 23.8% were male. Most of the participants were clinical students (59.7%) and 40.3% were preclinical students. Majority of the participants (55.8%) attended hybrid mode of study, 32.9% attended online learning sessions, and 11.3% attended face to face mode of study, respectively. 58.4% participants lived on campus, 24.9% lived at home and 16.7% were living alone or with friends.

**Table 1:** Sociodemographic information of respondents

Variable	Frequency	
	n	Percentage (%)
*Dental schools*		
IIUM	161	45.6
AIMST University	49	13.9
UM	40	11.3
UiTM	34	9.6
USIM	21	5.9
PIDC	16	4.5
IMU	6	1.7
MAHSA University	6	1.7
SEGi University	6	1.7
USM	5	1.4
UKM	4	1.1
MMMC	3	0.8
Lincoln University College	2	0.6
*Gender*		
Female	269	76.2
Male	84	23.8
*Age*		
< 20	4	1.2
20–25	338	95.6
> 25	11	3.2
*Year of study*		
Year 1	54	15.3
Year 2	88	25.0
Year 3	46	13.0
Year 4	119	33.7
Year 5	46	13.0
*Citizenship*		
Malaysian	348	98.6
Non-Malaysian	5	1.4
*Current mode of study*		
Hybrid	197	55.8
Online learning	116	32.9
Face to face	40	11.3
*Living condition*		
Staying at campus	206	58.4
Living at home, with family	88	24.9
Living alone/with friends	59	16.7

### Assessment of mental health concerns

As shown in Table 2, 72.8% respondents were anxious about their future and more than half (72.7%) felt anxious due to the uncertainty of how long the COVID-19 pandemic would last. Most of the participants (78.8%) participants were stressed, with almost half (69.1%) feeling angry due to not being in control of the current situation.

**Table 2:** Assessment of mental health

In light of COVID-19, how often did you:	*1 n (%)	*2 n (%)	*3 n (%)	*4 n (%)	*5 n (%)	Mean ± SD
Feel anxious about your future?	32 (9.1)	64 (18.1)	88 (24.9)	77 (21.8)	92 (26.1)	3.38 ± 1.291
Feel stressed?	18 (5.1)	57 (16.1)	128 (36.3)	92 (26.1)	58 (16.4)	3.33 ± 1.087
Feel anxious regarding the uncertainty about how long current crisis will last?	30 (8.5)	66 (18.7)	117 (33.1)	75 (21.2)	65 (18.4)	3.22 ± 1.196
Feel you could not cope with all of the things that you had to do?	27 (7.6)	76 (21.5)	114 (32.3)	77 (21.8)	59 (16.7)	3.18 ± 1.174
Feel angry because things were outside of your control?	30 (8.5)	79 (22.4)	134 (38.0)	71 (20.1)	39 (11.0)	3.03 ± 1.100
Feel you were unable to control important things in your life?	38 (10.8)	85 (24.1)	119 (33.7)	64 (18.1)	47 (13.3)	2.99 ± 1.179
Feel depressed?	81 (22.90)	88 (24.9)	96 (27.2)	56 (15.9)	32 (9.1)	2.63 ± 1.248
Feel lonely?	77 (21.80)	98 (27.8)	95 (26.90)	51 (14.4)	32 (9.1)	2.61 ± 1.229

1 (Not concerned at all) 5(Extremely concerned) *1 (Never) 5(Always)

### Assessment of physical health concerns

Table 3 summarizes the physical health concerns of dental students in the COVID-19 pandemic in which 76.5% respondents were concerned about their physical health. Most of the respondents (79.2%) were concerned about being infected with COVID-19 from providing patient care in the clinic compared to attending non-clinical exercises in schools (72.8%). Furthermore, 68.2% respondents felt anxious about being infected with COVID-19 from attending and interacting with friends at the respective schools.

**Table 3:** Assessment of physical health

In light of COVID-19, are you concerned about	1 n (%)	2 n (%)	3 n (%)	4 n (%)	5 n (%)	Mean ± SD
Getting infected by COVID-19 from providing patient care in the clinics?	26 (7.4)	47 (13.3)	82 (23.2)	101 (28.5)	97 (27.5)	3.56 ± 1.228
Your physical health?	20 (5.7)	63 (17.8)	102 (28.9)	88 (24.9)	80 (22.7)	3.41 ± 1.182
Getting infected by COVID-19 from interacting with people in the school buildings?	25 (7.1)	63 (17.8)	93 (26.3)	88 (24.9)	84 (23.8)	3.41 ± 1.226
Getting infected by COVID-19 from attending classes/performing preclinical/laboratory exercises in school?	29 (8.20)	67 (19.0)	88 (24.9)	92 (26.1)	77 (21.8)	3.34 ± 1.240

**In light of COVID 19, how often did you**	*1 n (%)	*2 n (%)	*3 n (%)	*4 n (%)	*5 n (%)	Mean ± SD
Feel anxious that you might be infected with COVID-19?	26 (7.4)	88 (24.4)	144 (40.8)	75 (21.2)	22 (6.2)	2.95 ± 1.000

1 (Not concerned at all) 5(Extremely concerned) *1 (Never) 5(Always)

### Assessment of academic concerns

In the assessment of academic concerns among dental students in the pandemic (Table 4), majority (91.8%) were concerned about the amount of clinical skills they would be able to acquire, followed by more than half (89.0%) were concerned about passing their dental professional exam in a timely manner. 86.4% respondents were concerned about completing their degree program on time. Most students (85.8%) suffered from lack of motivation in their studies, followed by difficulties focusing on work (80.2%). 80.2% respondents were willing to make up for the loss in their education experience by cancelling travel and 69.7% respondents were willing to take a shorter semester break after school re-opened to graduate on time.

**Table 4:** Assessment of academic performance

In light of COVID 19, are you concerned about	1 n (%)	2 n (%)	3 n (%)	4 n (%)	5 n (%)	Mean ± SD
The amount of clinical skills that you acquired?	4 (1.1)	25 (7.1)	57 (16.1)	92 (26.1)	175 (49.6)	4.16 ± 1.010
Passing your dental professional exams in a timely manner?	6 (1.7)	31 (8.8)	75 (21.2)	93 (26.3)	148 (41.9)	3.98 ± 1.067
The likelihood that you will complete your degree program in time?	11 (3.1)	37 (10.5)	86 (24.4)	95 (26.9)	124 (35.1)	3.80 ± 1.122
The quality of online courses?	6 (1.7)	42 (11.9)	92 (26.1)	106 (30.0)	107 (30.3)	3.75 ± 1.065

In light of COVID 19, how often did you	*1 n (%)	*2 n (%)	*3 n (%)	*4 n (%)	*5 n (%)	Mean ± SD
Feel difficult to find motivation in study?	8 (2.3)	43 (12.2)	89 (25.2)	101 (28.6)	112 (31.7)	3.75 ± 1.097
Feel difficult to focus on your work?	14 (4.0)	56 (15.9)	122 (34.6)	92 (26.1)	69 (19.5)	3.41 ± 1.092

In light of COVID-19, would you be willing to make up for the loss of education experience so that you can graduate on time	**1 n (%)	**2 n (%)	**3 n (%)	**4 n (%)	**5 n (%)	Mean ± SD
By cancelling all of your travels this year?	24 (6.8)	46 (13.0)	120 (34.0)	77 (21.8)	86 (24.4)	3.44 ± 1.186
By taking a shorter semester break after the school re-opens?	40 (11.3)	67 (19.0)	107 (30.3)	90 (25.5)	49 (13.9)	3.12 ± 1.202
By attending school 6 days per week after the school re-opens?	76 (21.5)	93 (26.3)	102 (28.9)	49 (13.9)	33 (9.3)	2.63 ± 1.227

1 (Not concerned at all) 5(Extremely concerned) *1 (Never) 5(Always)**1 (Not at all) 5(Definitely)

### Assessment of financial concerns

Table 5 presents the assessment of financial concerns in dental students, where 83.0% respondents feeling anxious about difficulties finding a job after graduation, followed more than half (77.0%) of the respondents were concerned about financial stability and being unable to cover their expenses in the next few months (64.6%).

**Table 5:** Assessment of financial health

In light of COVID-19, how often did you feel	*1 n (%)	*2 n (%)	*3 n (%)	*4 n (%)	*5 n (%)	Mean ± SD
Anxious that you will have difficulty finding a job after graduation?	14 (4.0)	46 (13.0)	101 (28.6)	110 (31.2)	82 (23.2)	3.57 ± 1.101
Anxious that you will be unable to cover your expenses in next few months?	47 (13.3)	78 (22.1)	96 (27.2)	78 (22.1)	54 (15.3)	3.04 ± 1.261

In light of COVID-19, are you concerned about	**1 n (%)	**2 n (%)	**3 n (%)	**4 n (%)	**5 n (%)	Mean ± SD
Your financial stability?	16 (4.5)	65 (18.4)	107 (30.3)	81 (22.9)	84 (23.8)	3.43 ± 1.168

*1 (Never) 5(Always) **1 (Not concerned at all) 5(Extremely concerned)

### The impact of COVID-19 on mental health concerns, physical health concerns, financial concerns and academic performance according to sociodemographic background

The impact of COVID-19 on mental health, financial concern and academic performance of dental students were compared according to their socio-demographic factors (Table 6). The mental, financial and academic concern of dental students significantly differ according to their year of study. It was found that Year 3 students shows the highest concern in these three domains.

**Table 6:** The impact of COVID-19 on mental health concerns, physical health concerns, financial concerns and academic performance according to sociodemographic background

Variables	N	Mental health concern score (Mean ± SD)	Physical health concern score (Mean ± SD)	Financial concern score (Mean ± SD)	Academic performance score (Mean ± SD)
*Gender*^a^					
Male	84	24.71 ± 7.910	13.12 ± 4.661	10.27 ± 2.889	15.40 ± 3.976
Female	269	23.29 ± 7.764	13.90 ± 4.310	9.96 ± 2.909	15.79 ± 3.647
*p*-value		0.160	0.163	0.526	0.558
*Year of study*^b^					
Year 1	54	24.67 ± 6.849	13.70 ± 4.542	9.24 ± 2.855	15.69 ± 3.185
Year 2	88	21.10 ± 7.154	13.95 ± 4.520	9.33 ± 2.827	14.65 ± 3.989
Year 3	46	28.20 ± 8.609	14.24 ± 4.557	10.96 ± 2.928	15.54 ± 4.043
Year 4	119	24.24 ± 7.694	13.89 ± 4.155	10.42 ± 2.901	16.21 ± 3.705
Year 5	46	24.37 ± 7	12.28 ± 4.375	10.41 ± 2.663	16.54 ± 3.153
*p*-value		0.000	0.236	0.001	0.022
*Current mode of study*^b^					
Face to face	40	24.47 ± 8.491	13.38 ± 4.918	10.05 ± 2.943	15.25 ± 4.573
Hybrid	197	24.99 ± 7.857	13.59 ± 4.346	10.26 ± 2.796	15.89 ± 3.583
Online learning	116	23.28 ± 7.675	14.03 ± 4.330	9.65 ± 3.048	15.52 ± 3.653
*p*-value		0.117	0.585	0.159	0.621
*Living condition*^b^					
Living alone/with friends	59	24.34 ± 8.446	13.68 ± 4.580	10.17 ± 3.069	15.68 ± 4.006
Living at home, with family	88	24.77 ± 7.477	13.43 ± 4.505	9.58 ± 3.020	15.60 ± 3.771
Staying at respective campus	206	24.21 ± 7.927	13.84 ± 4.321	10.19 ± 2.796	15.74 ± 3.641
*p*-value		0.809	0.839	0.284	0.958

^a^Mann-Whitney U test

^b^Kruskal Wallis test

## Discussion

COVID-19 has indeed affected dental students in various ways. Before the pandemic, commonly identified dental school stressors that placed dentistry as a stressful profession include extensive coursework, pressure to perform well, learning clinical procedures, and dealing with difficult patients [5, 9]. In addition to the usual stressors, dental students now face a global health crisis, institution closure, and challenges in practicing and honing their clinical skills. There is also an increased worry regarding dental protocols after the reopening of school, institutional responses to the pandemic, academic concerns, both physical and mental health concerns, and financial concerns which have added to the list of dental students stressors [5].

This study examined the impact of COVID-19 on dental students in Malaysia. Four main concerns were highlighted: physical health, mental health, financial health, and academic concerns. Based on our findings, year of study was the most important demographic criteria leading to outcomes. However, other demographic details such as gender, mode of study and living conditions have minor contributions to the result outcomes (Table 6).

A noteworthy point in accordance with our results in regards to the year of study and impact of COVID-19 was observed in Year 3 dental students. Year 3 students demonstrated a remarkable apprehensiveness towards the COVID-19 crisis, expressing the highest concerns compared to peers in other years of study. Mental and financial health concerns, as well as academic performance concerns were especially high. This may be explained by the lack of clinical exposure in Year 3 students compared to Year 4 and 5 students. Alzahem et al. disclosed that examinations and fear of failure were major stress contributors in pre-clinical students, whilst in clinical students the main stressor was clinical training, particularly factors related to finishing the clinical requirements [10]. Dental students face their first year in clinical practice in Year 3, and must deal with (1) transition from pre-clinical to clinical setting, (2) the shift from face to face and hands-on to online learning as a consequence of the global pandemic. Evidently, this situation proved difficult for the students to cope with. Additional restrictions caused by the pandemic meant that some students were not able to meet the lab requirements of some pre-dental courses due to institutional closure during Movement Control Order (MCO). This further caused fear in Year 3 students to go through their clinical years.

Students were generally concerned about their mental health; feelings of stress and anger were predominant due to lack of control over the current situation. Previous studies have reported that stress in dentistry students varied according to their ethnic differences, gender, and year of study [11]. In this study, it was found that female students experienced more stress compared to their male counterparts, supporting previously reported findings [11, 12] (Result not shown). Females generally express their emotions to a greater extent than males do, which may be heightened by the pandemic [2]. Studies indicate that females have a lower uncertainty tolerance compared to males and are subject to less coping strategies in times of uncertainty and stress, while males are more likely to avoid dealing with their emotions (avoidant coping) [2, 12]. On the contrary, cross-sectional studies done on medical students in India proved no significant difference in depression, anxiety, and stress between the groups of demographic variables during COVID-19 including gender, year of study, current residence, or family monthly income [13].

Approximately 30% of the students were extremely concerned about getting infected by COVID-19 from their patients and transmitting the virus to their family members at home (Table 3). Since dental practice exposes practitioners to saliva, blood, and other bodily fluids, dentists face an increased risk of infection by the coronavirus from suspected/confirmed COVID-19 patients. Additionally, they carry an increased risk of transmitting this infection to their peers, families, and other patients [5, 14]. A study by Ahmed et al. [15] reports that dentists have a genuine fear of carrying infections from their dental practices to their families and this may also contribute to the worry of the students on their family members’ well-being.

Since the Movement Control Orders (MCO) and Standard Operating Procedures (SOP) were implemented by the Malaysian government, there has been a shift from physical classroom teaching to online teaching due to the closure of public and private education institutions at all levels nationwide. Almost half of the students (41.6%) stayed at home and did not return to their institutions after school break [4, 16]. Our findings show that students were apprehensive with respect to the quality of their online courses (Table 4). In contrast, Hung et al. [5] reported positive student feedback towards their online curriculum. Besides the online curriculum, Malaysian university students also faced challenges with the technological infrastructure required for online learning. Poor internet connection was cited to be a main hindrance to effective online learning [2]. Upgrade and improvement of internet services is required to help resolve this problem. Government and telecommunication companies should play a role in expanding high speed internet services across the country. The increasing usage of mobile phones to about 6–8 h daily for attending online classes, high expectations from lectures, multiple assignments and non-flexible deadlines have contributed to insurmountable stress and health issues as stated by Sundarasen et al. [2].

The acquisition of adequate clinical skill time was the major concern among the dental students in this study, similar to findings by Hung et al. [5]. As a matter of fact, many expressed anxiety about simply being able to complete their degree or pass the dental board exams. Closing of institutions including the dental clinic, enforcement of strict standard procedures and limited number of patients during the pandemic translates into decreased learning time and less opportunities for clinical skill practice. Hands-on laboratory and clinical skills teaching have been disrupted during the COVID-19 pandemic and has been replaced with online learning. Unfortunately, this was proven to be ineffective at honing the students’ necessary psychomotor skills [17]. To improve online dental learning, some strategies were implemented, including simulation, demonstration, tele-dentistry, and videotaping cases with the faculty review. Sharing recorded videos of laboratory and clinical skills, in addition to online patient simulations or role plays is also beneficial [5, 17]. Fortunately, students were willing to cancel their travel plans, take shorter semester breaks and attend school 6 days per week in order to make up for the loss in education.

Moreover, many students reported lack of motivation and difficulty focusing on their studies following the pandemic (97.7% and 96% respectively) (Table 4). The sudden switch from in-person to online learning dental education forced students to adapt to a new way of learning and teaching [18]. A study by Ahmed et al. [15] on Malaysian postgraduate medical physics students, showed that students still favoured face-to-face compared to virtual learning modes. Compared to physical lecture sessions, the students found e-learning to be boring, less engaging and prevented them from effectively asking questions during lectures. In addition to loss of motivation and difficulty focusing on studies, the students also reported a drop in morale and internet connectivity problems as they need to adjust to new learning norms that require more independent and self-regulating learning approaches [15]. This can be improved by early introduction of online learning to the students, especially at the beginning of dental school enrolment and the expectations of virtual learning rather than face to face education should be made clear by explaining to the dental students in the course description along with reviewing current programmes and requirements related to the resources and infrastructure. Creativity of dental lecturers are required in order to make the students more eager and engage in online learning [19, 20].

Our findings also highlighted the financial concerns of dental students. Clinical students especially were anxious of being unable to cover their expenses in next few months (Table 5). Most students (96%) despite their year of study, were anxious that finding a job after graduating would be difficult, since unemployment is currently at its highest level since the depression era [5]. Based on a study in Malaysian university students, students were concerned with their ability to manage educational financial commitments due to family loss of income, loss of work opportunities and the possible need to self-finance their studies [2]. This finding is identical to reports by Hung et al. [5]. Hung et al. [5] further reports that students closest to finishing their studies and seeking jobs, i.e. senior dental students and residents (final year students in our study) expressed the most concern. Dentistry has the highest debt-to-income ratio, which may cause dental students who have invested many years into their education to face looming debt and unemployment. As the effects of the COVID-19 pandemic are felt by Malaysian dental students, further research is advised for the inclusion of successful coping strategies for students during this uncertain and testing time. We hope that our findings of this study could assist dental colleges and universities in Malaysia in determining the dental school stressors and identifying evidence-based intervention practices to aid the students in times of similar pandemic in the future. It should also provide guidelines for policymakers on possible mechanisms to moderate the academic, financial, mental and physical impacts on dental students during such health crises.

This study does present with its own limitation. The result of this study may not represent all Malaysian’s dental students. Inability to reach all dental students from exactly 13 dental schools leads to disproportionate number of respondents presenting each dental school, thus may increase the risk of bigotry. In this study, data was collected in the months of March and April 2021 when most of the students had already being allowed to attend school after the closure of institutions during MCO. The concerns of the students could have been varied if the data had been collected at the peak of COVID-19, which was at the end of year 2020. Moreover, there is always uncertainty of whether the respondents answered the questions honestly when it comes to surveys with questionnaires.

## Conclusions

The COVID-19 pandemic significantly impacted dental education. Our findings indicate that students are generally concerned about their mental health, financial health and academic performance. Half students appear apprehensive with respect to the quality of their online courses and most of them have lacking motivation and difficulty focusing on their studies following the pandemic. This study provides meaningful insights to inform decisions not only for dental schools but also for all health professional schools and the public.

## Data Availability

Data of the study will be shared upon request to the corresponding author.
